# Practicing provider-initiated HIV testing in high prevalence settings: consent concerns and missed preventive opportunities

**DOI:** 10.1186/1472-6963-11-87

**Published:** 2011-04-21

**Authors:** Mercy K Njeru, Astrid Blystad, Elizabeth H Shayo, Isaac K Nyamongo, Knut Fylkesnes

**Affiliations:** 1 Centre for Public Health Research, Kenya Medical Research Institute, Nairobi, Kenya; 2 Centre for International Health, Faculty of Medicine and Dentistry, University of Bergen, Bergen, Norway; 3 Departments of Public Health and Primary Health Care, Faculty of Medicine and Dentistry, University of Bergen, Bergen, Norway; 4 National Institute for Medical Research, Dar Es Salaam, Tanzania; 5 Institute of Anthropology, Gender and African studies, University of Nairobi, Nairobi, Kenya

## Abstract

**Background:**

Counselling is considered a prerequisite for the proper handling of testing and for ensuring effective HIV preventive efforts. HIV testing services have recently been scaled up substantially with a particular focus on provider-initiated models. Increasing HIV test rates have been attributed to the rapid scale-up of the provider-initiated testing model, but there is limited documentation of experiences with this new service model. The aim of this study was to determine the use of different types of HIV testing services and to investigate perceptions and experiences of these services with a particular emphasis on the provider initiated testing in three selected districts in Kenya, Tanzania, and, Zambia.

**Methods:**

A concurrent triangulation mixed methods design was applied using quantitative and qualitative approaches. A population-based survey was conducted among adults in the three study districts, and qualitative data were obtained from 34 focus group discussions and 18 in-depth interviews. The data originates from the ongoing EU funded research project "REsponse to ACountable Priority Setting for Trust in Health Systems" (REACT) implemented in the three countries which has a research component linked to HIV and testing, and from an additional study focusing on HIV testing, counselling perceptions and experiences in Kenya.

**Results:**

Proportions of the population formerly tested for HIV differed sharply between the study districts and particularly among women (54% Malindi, 34% Kapiri Mposhi and 27% Mbarali) (p < 0.001). Women were much more likely to be tested than men in the districts that had scaled-up programmes for preventing mother to child transmission of HIV (PMTCT). Only minor gender differences appeared for voluntary counselling and testing. In places where, the provider-initiated model in PMTCT programmes had been rolled out extensively testing was accompanied by very limited pre- and post-test counselling and by a related neglect of preventative measures. Informants expressed frustration related to their experienced inability to 'opt-out' or decline from the provider-initiated HIV testing services.

**Conclusion:**

Counselling emerged as a highly valued process during HIV testing. However, counselling efforts were limited in the implementation of the provider-initiated opt-out HIV testing model. The approach was moreover not perceived as voluntary. This raises serious ethical concerns and implies missed preventive opportunities inherent in the counselling concept. Moreover, implementation of the new testing approach seem to add a burden to pregnant women as disproportionate numbers of women get to know their HIV status, reveal their HIV status to their spouse and recruit their spouses to go for a test. We argue that there is an urgent need to reconsider the manner in which the provider initiated HIV testing model is implemented in order to protect the client's autonomy and to maximise access to HIV prevention.

## Background

HIV counselling and testing (HCT) services are a crucial part of prevention and are necessary as a prerequisite for supporting, caring and providing treatment to the HIV infected. Preventive counselling gives individuals an opportunity to receive relevant information, to correct misconceptions about HIV, to assess risk and to motivate behaviour change if necessary [1,2]. Preventive effects of the voluntary counselling and testing (VCT)-package (risk-reduction counselling in relation to testing) are reasonably well documented [3,4], but to what extent knowledge of ones own status alone leads to behaviour change is difficult to address in ethically acceptable research efforts. HCT also has further been noted to have the potential to encourage openness, hence contributing to the reduction of fear and stigma in society [1,2,5]. Despite the assumed benefits and scaling-up of HCT services over the last decade the demand for the services has been disappointingly low in countries with the most serious epidemics [6-9]. This has resulted in efforts to establish alternative HIV testing models such as the provider-initiated (PITC) model, which differs substantially from the established client-initiated model.

Client-initiated HIV counselling and testing is commonly referred to as voluntary counselling and testing (VCT). It is motivated by an individual's right to know his or her HIV status, and it takes place when an individual seeks counselling and testing at a facility that offers the services. This type of HIV testing and counselling service is offered widely, mainly through facilities integrated in health settings, mobile services and in stand-alone facilities that are located away from health care facilities such as community-based settings and night services [5,9-15]. Provider-initiated testing in contrast to the client initiated testing is recommended by a health provider to people attending a health facility [15]. This approach to testing can be offered with an opt-in or an opt-out approach, the latter being prominent in specialized programmes such as prevention of mother-to-child-transmission programmes (PMTCT) [15]. The difference between the two is that with the opt-in approach patients need to affirmatively agree to test before the test is conducted, whereas in opt-out approaches clients must actively decline after the pre-test information is offered, if they do not want the test to be performed [15]. Offering testing to all individuals seeking health care services is assumed to increase HIV test rates and thus improve access to treatment. HIV testing has been recommended for all pregnant women, but with a preservation of the woman's right to decline or opt- out. Those who decline are however encouraged to undergo HIV testing at a subsequent visit [15].

The new guidelines recommend pre-test information in place of pre-test counselling when practicing PITC [15]. However, critics argue that individuals tested for HIV must be allowed to evaluate the information provided to them during individual pre-test counselling sessions, and come to their own conclusion regarding whether or not they wish to be tested [16,17]. This is deemed vital for ensuring trust in the health services not the least in the prevention, care and treatment of HIV. A trusting relationship between the health care provider and patient (interpersonal trust) within the context of HIV testing has been seen as essential in enhancing the quality of the interaction and for constructively encouraging necessary behaviour change [18].

The increase in HIV test rates documented in certain settings has been partly attributed to a rapid scale-up of provider-initiated testing services [19-21]. However, the roll-out of this testing model has been criticised from an ethical and human rights perspective for paving the way to neglect of informed consent [16,17,22], and for reducing the amount of counselling that accompanies the HIV test [15,23]. Despite the early voicing of ethical and human rights concerns related to the provider-initiated model of HIV testing, there is little empirical evidence from Africa related to client experiences with this model of testing. This study is an attempt to explore the perceptions and experiences of the testing services with a particular emphasis on the provider initiated opt-out HIV testing as practised at Antenatal Clinics (ANC) where the PMTCT programme is located. Moreover we investigated the exposure to HIV testing in the adult populations in three African districts: Malindi, Kenya; Mbarali, Tanzania; and Kapiri Mposhi in Zambia, where the EU-funded multi-country project, REACT (Response to A Countable priority setting for Trust in health systems) is currently implemented. The project draws upon the ethical framework 'Accountability for Reasonableness' (AFR), for legitimate and fair priority setting. It is applied to provide decision makers with guidance to enhance trust, quality and equity in health systems [24].

## Methods

### Study design

**Figure 1:**
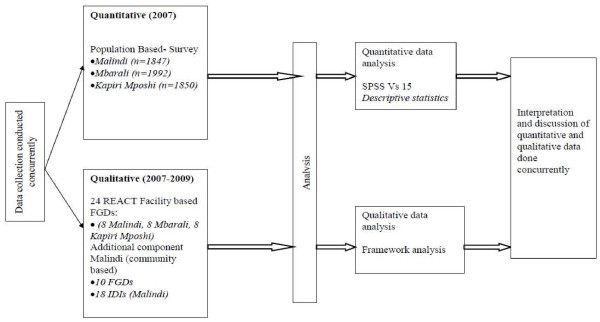
**Study design**.

A mixed method approach was applied in which quantitative and qualitative data were collected using a concurrent triangulation design [25] as illustrated in Figure 1. Quantitative methods were used to determine the proportions of people undergoing HIV tests and the proportions utilizing the various HIV testing services. The qualitative in-depth interviews (IDIs) and focus group discussions (FGDs) were employed to explore informants' experiences and perceptions of the HIV testing services with an emphasis on experiences with the provider initiated testing model.

### Study area and population

The study was carried out at three different study sites, namely: Malindi district in Kenya, Mbarali district in Tanzania and Kapiri Mposhi district in Zambia. The districts were selected on the basis of assumed similarities [24], but closer look at the health statistics reveals differences in key indicators, among them HIV prevalence. HIV control programmes were defined as one of several evaluation domains of the REACT project. The national HIV prevalence levels within these countries show marked geographical variation; the overall national levels for adults aged 15-49 years have been estimated at 7.7% in Kenya [26], 6.2% in Tanzania [27] and 15.2% in Zambia [28]. In the selected study districts the HIV prevalence's are estimated at: 16% in Malindi district [29], 7.9% in Mbarali district [30], and 13% in rural and 32.2% in urban Kapiri Mposhi district [31,32]. Malindi has an estimated population of 350,000 [29,33], Mbarali 235,000 [30] and Kapiri Mposhi 300,000.

### Quantitative study

The quantitative study conducted in 2007 comprised a population-based survey that identified household members at community level in the three districts as part of the REACT project. A stratified random cluster sampling technique was employed with stratification by district and urban vs. rural populations. The intended sample size was 2000 in each district. The samples were selected from the national statistical master sample frames in all three districts. The steps in sampling were random selection by probability proportional to size of enumeration areas at the strata (clusters); random selection of an equal number of households within each of the randomly selected clusters; and random selection of one male and one female from each household as study participants.

### Data collection and analysis

A questionnaire was administered in English in Kapiri Mposhi and Swahili in Malindi and Mbarali. Socio-demographic measures included age, residence, sex, marital status, educational attainment and the times of visits to different types of health facilities during the previous 12 months. Other measures included previous HIV testing and the type of testing (e.g. client-initiated VCT or provider-initiated PMTCT). Data were entered in Epi-info and analysed using SPSS version 15 for Microsoft Windows. The Pearson chi-square test was used to evaluate differences between groups regarding proportions of HIV test exposure and socio-demographic characteristics. P-values below 0.05 were considered statistically significant.

### Qualitative study

The qualitative data set originates from two sources. The first set consists of 24 FGDs (eight from each country) conducted in 2007 in Mbarali and in 2008 in Malindi and Kapiri Mposhi as part of the REACT project. These FGDs were carried out among individuals seeking health services at health care facilities that served the sampled clusters. The second data set consists of 10 FGDs and 18 in-depth interviews (IDIs) conducted in one of the study districts (Malindi) in February 2009. The FGDs in both data sets explored the practices, ideas and experiences related to HIV testing models. The IDIs explored more thoroughly experiences and perceptions related to the provider initiated opt-out strategy.

### Recruitment of informants and data collection

Participants and the health facilities in the first set of 24 FGDs were recruited purposely. The groups consisted of: female out-patients, male out-patients, pregnant women attending ante-natal clinics and youths aged 18 to 24 years. Questions developed for use in the population-based survey guided the development of the topic guides used for this set of FGDs. The FGD's were conducted by trained social scientists. In the second phase of data collection, in the sub study carried out in Malindi, 10 FGDs and 18 IDIs were conducted at community level. The age of the FGD participants ranged between 18 years and 68 years while that of the IDI participants ranged between 18 to 50 years. Informants for these group discussions and interviews were recruited in urban and rural settings among five categories of informants: female youths, female adults, male youths, male adults and pregnant women. The interviews in the second phase of data collection were conducted by the first author (MKN) with assistance from an experienced social scientist with post-graduate training.

For each FGD (in both data sets) and IDI socio-demographic data on age, marital status, level of education, occupation and spouse occupation were recorded. The purpose of the meeting and the main themes for discussion were introduced by the moderator prior to the start of the sessions. Participants were given the chance to discuss the given themes thoroughly and emerging ideas were followed up with further questions. Observations of the group dynamics during discussions were recorded by an assistant. The FGDs lasted 2 to 3 hours while the IDIs lasted between 1 and 2 hours. All the conversations were audio-taped.

The contents of the FGDs and IDIs were first transcribed verbatim and carefully translated to English - within the same document - with emphasis on retaining the meaning of the culturally-embedded concepts and expressions. 'Framework analysis', a recognised framework for applied qualitative research was employed. Data analysis for all the data sets involved five main steps: familiarization, identification of a framework, indexing, charting and interpretation [34,35]. The main analysis was carried out manually by four of the authors who speak Swahili (MKN, AB, EHS and IKN). Familiarization with the data implied immersion in the raw data several times through listening to the tapes and reading the full sets of transcripts. A framework was developed from emerging issues identified during the familiarization stage. The main emerging themes were related to what was experienced as the value of counselling and the challenges experienced with the implementation of the PITC model of HIV testing by the study participants. These themes were further explored through a thorough and time consuming review of each transcript. Codes were written on the margins of the transcripts leading to the development of a chart matrix. The chart was thoroughly discussed and interpreted in meetings among all the co-authors.

#### Ethical aspects

Ethical clearance for the umbrella project (REACT) was obtained from research clearance organizations in the three countries prior to the study; in Kenya scientific and ethical approval was obtained from the Kenya Medical Research Institute (KEMRI) and from the National Ethical Review Committee (NERC) of Kenya. In Tanzania research clearance was obtained from the Medical Research Coordinating Committee (MRCC) of the National Institute of Medical Research (NIMR), and in Zambia from the University of Zambia Research Ethics Committee. Written informed consent was obtained from all participants of the population-based survey prior to the interviews, and oral consent was obtained for the FGDs and IDIs. Confidentiality and anonymity of the study informants were emphasised and maintained throughout the study.

## Results

### Participation in the survey

A total of 6088 persons were sampled, and the achieved sample size was almost equal among district and urban/rural strata except for Malindi where the rural stratum was over-sampled. The overall response rate was 93.4% (n = 5689) and non-response did not differ by district.

### Population characteristics and health care use

The project districts differed with regard to the educational attainment of the participants, and there was a particular contrast between Mbarali and the other two districts; e.g. in the urban population the proportion with nine or more years in school was 9.7% in urban Mbarali, compared to 48% in urban Malindi (p < 0.001) and 44.8% in Kapiri Mposhi (p < 0.001. Malindi and Kapiri Mposhi were characterised by a marked differential in the level of education in general, and with inequalities between the urban and rural populations in particular; e.g. the proportions of urban versus rural populations with nine or more years of schooling were 48% versus 16.4% in Malindi (p < 0.001) and 44.8% versus 22.6% in Kapiri Mposhi (p < 0.001). In contrast, the population in Mbarali appeared to have small attainment differentials, e.g. more than 70% spent 5-8 years in school and there were no urban/rural differentials (Table 1). There was a higher likelihood of being married in the rural populations in the three countries. Self-reported health care use among the survey participants indicated that the health care system was solely public in Kapiri Mposhi, in contrast to the situation in Malindi where there was substantial use of private facilities and in Mbarali where there was a mixture of public, private and faith-based facilities.

**Table 1:** Socio-demographic characteristics of the respondents

*Characteristics*	*Malindi N = 1847*				*Mbarali N = 1992*				*Kapiri Mposhi N = 1850*			
**Residence**	**Urban**		**Rural**		**Urban**		**Rural**		**Urban**		**Rural**	

	**%**	**n**	**%**	**n**	**%**	**n**	**%**	**n**	**%**	**n**	**%**	**n**

**Age**												
15-19	10.9	(68)	15.5	(187)	12.4	(116)	7.6	(80)	12.1	(111)	11.7	(109)
20-24	23.9	(149)	16.1	(194)	18.7	(175)	18.4	(195)	18.1	(166)	15.5	(144)
25-29	21.2	(132)	16.8	(203)	19.9	(186)	20.9	(221)	20.0	(184)	19.5	(182)
30-39	30.3	(184)	30.6	(369)	32.2	(301)	36.3	(384)	33.2	(302)	32.4	(302)
40-49	13.8	(88)	21.0	(253)	16.8	(157)	16.7	(177)	16.2	(148)	20.8	(194)
**Sex**												
Male	50.6	(318)	50.3	(613)	49.9	(467)	48.9	(517)	43.4	(399)	45.1	(420)
Female	49.4	(310)	49.7	(606)	50.1	(468)	51.1	(540)	56.6	(520)	54.9	(511)
**Marital status**												
Single	28.8	(180)	20.8	(252)	16.8	(157)	10.2	(108)	20.6	(189)	14.9	(139)
Ever Married	63.1	(394)	74.2	(899)	83.1	(777)	89.6	(947)	70.6	(647)	75.5	(703)
**Yrs of Educ**												
0 yrs	7.8	(49)	29.8	(363)	10.9	(102)	10.8	(114)	3.9	(36)	7.5	(70)
1-4 yrs	11.9	(75)	15.6	(190)	7.2	(67)	6.5	(69)	7.3	(67)	19.1	(178)
5-8 yrs	32.2	(202)	38.2	(466)	72.3	(676)	76.2	(805)	44.0	(404)	50.8	(473)
9-10 yrs	14.6	(92)	9.0	(110)	4.2	(39)	2.0	(21)	23.4	(215)	15.7	(146)
>11yrs	33.4	(210)	7.4	(90)	5.5	(51)	4.5	(48)	21.4	(197)	6.9	(64)

*Differences were calculated using Pearson chi square test

### Exposure to HIV Testing

**Figure 2:**
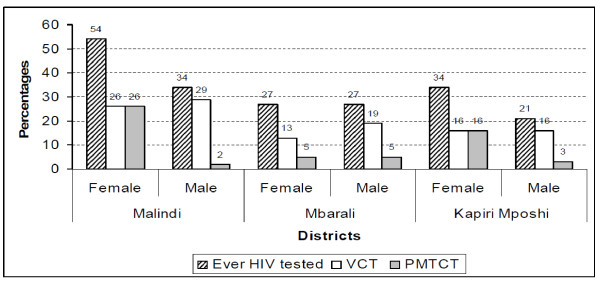
**Proportions testing for HIV test and proportions testing VCT and PMTCT**.

Close to a third (33.5%, n = 5649) of the respondents had been tested for HIV, but there were differences among districts (Malindi 44.2%, n = 1829; Mbarali 27.3%, n = 1982; Kapiri Mposhi 28.3%, n = 1838). Women were significantly (p < 0.001) more likely to have been tested than men (38%, n = 2936 vs. 28%, n = 2710). These differences between men and women were also reflected within the districts (Figure 2). Women in Malindi were more likely to have been tested than women in Mbarali (54%, n = 911 vs. 28%, n = 1004) and Kapiri Mposhi (54% vs. 34%, n = 1023) (p < 0.001), while women in Kapiri Mposhi were more likely to have been tested than women in Mbarali (Figure 2). Only minor gender differences were observed for VCT based testing, mainly in Mbarali district (Figure 2). In the Mbarali district, about 9% of the women indicated they had been tested in other places e.g. private clinics and hospitals. In the same district, the likelihood of having been tested did not differ by gender, and there was a relatively low coverage of PMTCT based testing. Malindi district had a notably higher testing rate than the other two regarding HIV tests through VCT (Figure 2).

#### Perceptions and experiences with counselling during HIV testing

In the following section findings from the focus group discussions and the in-depth interviews are presented.

#### The value of counselling in HIV testing

During the interviews, counselling was brought up by the informants as an essential and appreciated part of the HIV testing regimes. Both the prevention and the support aspects emerged as highly valued parts of these sessions.

### The preventive aspect

The preventive opportunities inherent in the counselling concept emerged as important in our interviews and discussions.

"*The importance of counselling emerges when a person who has not been infected gets advice and follows it, because s/he^1 ^will not get this disease." *(Female 26 years old, urban Malindi). *"Counselling is very important because if you are counselled you get the courage or the strength to prevent being infected as you are told the way forward." *(Female youth, FGD, urban Malindi)

Informants acknowledged that preventive counselling was practised when they underwent VCT.

*"As for me I was tested at the VCT. There they really counselled me on HIV and on how I can protect my life." *(Male adults, FGD, urban Malindi)

### The support dimension

The need for counselling as an important dimension in supporting those already infected with HIV was a point that emerged in both the IDIs and the FGDs across the three districts. Both male and female informants from the three countries expressed the view that sufficient post-test counselling has the potential to reduce worry, fear and blame as illustrated in the quotations below:

*"Because you may have come there for testing and if you are found (meaning: if you are found to be HIV positive) you will have a lot of worry because in your heart you wonder: 'now I have been found, now what will I do?' Then you have a lot of thoughts, but if you find a person that gives you counselling or ideas on what you will do, it will cool your heart. You will be able to settle a little bit." *(Male 43 years old, rural Malindi)

*"Because if you have been made aware through counselling, even if you tested positive, there will be no fear, that's why some people declare that they are HIV positive, they had seminars where they were counselled and that is why they have that courage. But if one discloses his status as positive here people will talk about him; some will even deny him drinking water because of fear" *(Male adults, FGD, Mbarali)

### The time dimension

The need for sufficient time during counselling in order to convey the messages clearly was experienced as vital in the testing services. More time was expressed as important in order to fully conceptualise and understand the information presented before embarking on the testing so that one would be prepared to receive the test results whatever the outcome might be.

*"When I say we need education, I mean we need counselling, we need counselling that is offered step by step until we are ready to test." *(Male adults, FGD, Mbarali)

"*Not that when you enter the facility and after the counselling you are immediately asked if you are ready for the test. No! That also can cause a lack of willingness to test. The counsellor should counsel me and tell me I have the right to go for testing. Therefore, if the time for counselling is increased, I see that as an improvement"*. (Female adult, FGD, rural Malindi)

Informants indicated that at the VCT service centres testing is only done after counselling and when the client is ready. This view is expressed for example by an informant in Kapiri Mposhi who noted:

"*When you go for VCT, you are counselled and after that, if you are ready to know your status, that's when you are tested." *(Male youth, FGD, Kapiri Mposhi.)

A female informant from Malindi echoed these views thus:

"*When you get to the VCT, first of all they offer counselling. You are asked many questions and you continue up to the end, and that is when you are tested*." (Female 50 years old, rural Malindi)

#### Challenges experienced with the implementation of the PITC model in HIV testing

The following section draws primarily on the findings from Malindi district.

The study findings indicate that in this district, where the provider initiated testing approach had been scaled up extensively, challenges were faced in the manner in which this new model was implemented.

### The threat to counselling

There were differences in the experiences related to HIV counselling at the ANC services in the three districts. In Malindi where the provider initiated opt-out HIV model had been scaled up extensively at the ANC clinics, informants expressed limited or lack of the pre-test counselling and post-test counselling was not present for HIV-negative individuals, as expressed in the following dialogue from Malindi:

• *Moderator: Do they counsel you before testing for HIV?*

• *Participant 2: They counsel you only when you are found to be HIV positive, but if you are not HIV positive you just get your results and go*. (Female pregnant, FGD, rural Malindi)

However, informants from Mbarali and Kapiri Mposhi reported the presence of both pre and post test counselling at the ANC clinics during HIV testing as presented below:

*"We were counselled and it was recommended that we test for HIV before becoming pregnant, but if we are pregnant already we also have to be counselled and tested; say you are found positive, you are to be given drugs to protect the child from infection during delivery." *(Female pregnant, FGD, Mbarali, Tanzania)

"*When we went for counselling we were taught. They started teaching us how someone can live positively and strongly. There were papers on the wall, and they showed us what a person looks like before HIV and after. And for us who were pregnant, we were taught how to take care of ourselves and how to protect the unborn baby (if found HIV positive), even during delivery." *(Female Out- patient adults, FGD, Kapiri Mposhi)

### HIV testing as Mandatory

In Malindi our informants reported that the HIV test within PMTCT was no longer voluntary. A common phrase that was used to describe the new testing model was "it is a must", a point noted by both female and male respondents:

*"I was not tested at a VCT centre, but at that place for women (ANC clinic). Because when you are pregnant, you are tested on many things, but first they must test you for AIDS." *(Female pregnant 40 years old, urban Malindi)

*"Here let's say women and men go for (HIV) testing, but a majority of them are women because the woman must be tested when she goes to the clinic." *(Male 34 years old, urban Malindi)

In Malindi, the study informants explained that little was done at the ANC station to prepare the women for the testing. As a result the women were taken by surprise and panicked when they understood that they were to be tested for HIV.

*"During the second pregnancy we were not given a choice. It was a must to get tested on HIV and then (after that) on the pregnancy. We were not asked; you enter in the room for HIV testing and then you go for other tests. To tell you the truth, some there got quite scared that day when we were suddenly tested. People panicked a lot. So people were not happy, but it was a must that they do it." *(Female 35 years old, urban Malindi

The large majority of our informants were disturbed by the lack of choice provision in the new provider initiated approach to the testing services. A few informants, who were from areas where the provider initiated opt-out approach had not been introduced, approved of the new approach and expressed the view that the testing should be mandatory.

 "*It should be made simple such that when you go to the district hospital for any reason, you should be tested. They should not wait for voluntary counselling. He/she should be tested and provided with life lengthening drugs. Why don't they offer testing services immediately? In my opinion as soon as a person goes there, there are no reasons for delaying him or her, he/she should be tested and if found positive then he/she should start using medication." *(Male, FGD, Mbarali Tanzania)

Despite the fact that the option to opt-out or decline a test is a part of the PITC model, our informants explained that it was in practice not possible to decline HIV testing at the ANC. Opting out implied that further care is declined.

*"It was said that according to the rules of the hospital if someone reaches the time of delivery and does not have HIV results she is not received." *(Female 35 years old, urban Malindi)

*"If you refuse to test they don't examine your stomach. So when it is time for delivery they don't accept you." *(Female pregnant, FGD, Urban Malindi)

M: They tell you they are testing you whether you like it or not?

*I: Whether you like it or not. If you choose to run away, where will you go? There is no need for running away because you will just have to come back*. (Female pregnant 40 years old, urban Malindi)

### The expressed burden on women

Many more women than men were tested for HIV as indicated by the population-based survey (Figure 2). This difference was more marked in Malindi district. Our informants described the burden that this places on the women. Another recurring theme was the difficulty of revealing information to partners that also they should go for testing. The burden was partly revealed through statements where our informants expressed the need to involve the health personnel in bringing their spouses to test as shown in the quote below.

*"These counsellors should be many to help us because we are wives, and when you ask your husband to go to test himself he stays quiet refusing to talk. He tells you 'you get tested, if you are found to be ok, I am also ok'. He does not go." *(Female pregnant, FGD, urban Malindi*)*

This finding was further confirmed by men in the male FGD discussion where men's attitudes towards spouses testing and further clarification on the challenges faced by the women on disclosure is illustrated in the following quote.

*'Let me say what men say to their wives, because of their mentality: "Now aren't you the one who has gone for testing and 'you are mine' isn't that right? You have been tested and you are ok? Now what problem do I have?" *(Male adult, FGD, rural Malindi)

The challenge that a disproportionately large number of women gets to know their HIV status implies numerous difficulties as the woman will be confronted with the fact that she is the one found to be HIV positive as the following quote reveals

*"You know also there are many incidents which have come up because you find that when a woman is heavy (pregnant) it's like the husband forces the wife to go for testing, you see? If anything bad arises (meaning if she is HIV positive) he starts questioning the wife, and asks 'where did it come from?'" *(Female pregnant, FGD, rural Malindi)

However in very few cases it was expressed that women too forced their husbands to test as expressed in the following quote

*"Mostly women are the ones who force men to go for testing when she is about to be married, or if a wife suspects her husband has another side marriage, she forces the husband to get tested"*. (Female 33 years old, urban Malindi)

Further there was blame towards the PMTCT program as illustrated in the following quote.

*"Now you can see that it is easy to be infected because at this moment when the young women start bearing children and visit the hospital, they have HIV testing done at the PMTCT centre. When they are found to be infected and when the woman returns home, there is a lot of 'stigma', because this is a young woman who has delivered, and it thus looks as if she is the one responsible for bringing the disease home, while it is her husband who came to infect her when she was expecting. You will note that this disease has kind of brought in other very difficult situations, especially in the areas of testing at the PMTCT." *(Female adult, FGD, rural Malindi)

There was a strongly expressed need to involve the men or husbands when pregnant women are tested.

*"For me, I would feel good if there was a way these men can also be forced to go for testing instead of waiting until their wives get pregnant." *(Female pregnant, FGD, rural Malindi)

The consequence of primarily testing women was experienced as severe.

*"It is important that a pregnant mother is tested along with the husband at the PMTCT, so that they can be counselled and come together for testing. Otherwise you even see many marriages breaking down. Therefore counselling both husband and wife is very important." *(Female adult, FGD, rural Malindi)

## Discussion

The population-based survey conducted in the selected study districts in the three countries revealed marked country differences in HIV testing exposures. These differences are primarily linked to differences in the roll-out of the provider-initiated opt-out approach to testing in the PMTCT programs at the ANCs.

Our findings indicate that the manner in which the provider initiated opt-out testing model is being interpreted and implemented has serious unintended negative effects. Firstly a reduced focus on counselling in general was found, and limited or no preventive counselling taking place for persons with HIV negative test results was particularly brought up. Secondly the provider-initiated opt-out strategy was perceived to impede the much-valued consent process inherent in pre-test counselling. The practice was experienced as an obstruction to choice as it was considered impossible to opt-out of the test. Thirdly, the scaling-up of the provider initiated opt-out model through PMTCT was found to result in striking gender differences in knowledge of personal HIV status with consequent burden on women. The survey revealed striking gendered differences between countries related to HIV testing, with a female to male ratio of 1.6 in Malindi and Kapiri Mposhi districts, the districts that presented a high exposure in HIV testing as part of PMTCT. No gender difference appeared in Mbarali district where the exposure to PMTCT was relatively low. The study revealed that the strategy was perceived to add an unreasonable burden on women who increasingly have to communicate the test results to their partners when they are pregnant as well as recruit them to go for testing.

**Figure 3:**
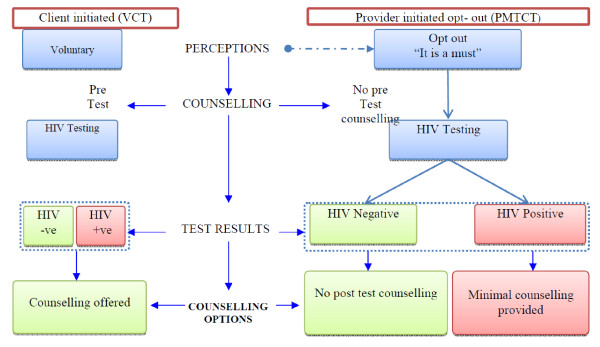
**The client initiated (VCT) and Provider initiated Opt- out (PMTCT) HIV testing process as experienced by our informants**.

The provider-initiated opt-out testing model has been described as successful in achieving higher numbers of individuals to test [21,36-39]. Our results indicate that substantially more women seem to have been tested in areas where this model had been scaled up. The provider initiated testing and counselling guidelines, recommend that post test counselling is performed after an HIV test [15]. On implementation, our results revealed however, that there was reduced focus on counselling during HIV testing at the ANC settings. The informants expressed the view that no or limited preventive counselling took place for persons with negative test results within the new approach, as summarised in Figure 3. The expansion of testing, which has been closely linked to rapidly-increasing financial support for treatment, has led many countries to shift their focus towards treatment with less focus on HIV prevention [40]. Post-test counselling for persons with a negative test result offers vital opportunities for prevention. It gives health care providers the opportunity to assess the degree of risk related to the client's lifestyle and enables him/her to define and communicate potential behaviour change and ways to sustain this behaviour change [2,41].

Despite the expressed importance of post-test counselling for prevention by our informants, the findings suggest that this was often lacking for people with negative test results. This is in our view an alarming development, and implies increasing numbers of missed opportunities for prevention, as some recent studies have indicated. A study from South Africa showed that pregnant women had four times the transmission risk of other women [42] indicating the importance of reaching this particular category with messages focusing on prevention of horizontal transmission. The increased sexual risk-taking that followed a negative test result in a cohort conducted in Zimbabwe [43] moreover indicates the potential dangers of leaving out individuals who have tested HIV negative without proper preventive counselling. In line with this argument a recent population-based open cohort study related to behaviour change associated with VCT showed substantial risk reduction, particularly among women, regardless of their HIV status [44].

The finding of limited pre-test counselling in our study is consistent with other publications that have raised the issue of neglected counselling within this testing model [10,16,22]. Counselling is one of the three Cs' (consent, confidentiality and counselling) perceived as core in HIV testing [45]. Given that counsellors are properly trained to assist in the process of attaining consent, pre-test counselling plays a fundamental role. It promotes the individual's autonomy by offering an opportunity to make an informed decision that is critical for HIV testing [2,41]. Individual autonomy is an important element of 'responsiveness'; a fundamental goal of health systems [46,47] and critically important in HIV testing [48].The implementation of the provider initiated opt-out testing model perceived as mandatory as reflected in the formulation " it is a must" by our informants, not only contradicts the current global guidelines, which emphasize the rights of clients to decline or opt-out of HIV testing when offered [15,45] but also national guidelines on HIV testing [5]. Similar concerns have been raised in Botswana. Even though high proportions were in favour of the opt-out testing model, a majority (68%) felt that it was not possible to refuse a test [49,50]. Studies and reports from Kenya and Uganda reveal that women seeking maternal care were not provided with the opportunity to give informed consent, nor counselled prior to or after testing [51-53].

Such scenarios have created an important discussion related to the classical dilemmas on how to balance public health benefits with the individual good. One major argument has been that the benefit of mandatory testing in preserving human life (as the HIV positive individual gets the opportunity to receive treatment) outweighs arguments related to human rights and autonomy [54]. This argument has gained grounds in the context of prevention of vertical transmission. It is in our view important to be aware of what is lost in the process when PITC models are not implemented with caution and with fundamental respect for the key principles on which it is based. The provider initiated opt-out testing has thus substantially increased access to prevention of vertical transmission of the infection [21,55], which was one of the main intentions behind the changes in testing approaches [2,56] in ANC settings. While there is increasing demonstration of success related to the provider initiated opt-out model in terms of testing coverage, we argue that the ways in which the PITC model is implemented calls for a consideration of aspects beyond the proportions exposed to testing and the proportions offered treatment.

Our findings on HIV testing exposure have furthermore indicated unforeseen inequity challenges emerging from the fact that with scaling up of the provider initiated opt-out testing in PMTCT programs; women are exposed to testing to a much larger extent than men. Previous studies have demonstrated that the mere act of being tested for HIV can be enough to subject a woman to domestic violence [51]. The wide recognition of potential adverse effects of disclosure to husbands adds seriously to the burden of knowing ones HIV status [16,57]. It has been demonstrated that women with HIV positive test results may be confronted with severe violence that includes physical and verbal abuse also from staff at the health facilities [51]. In Kenya such challenges have led to the development of a tribunal that aims to handle legal HIV-related issues including discrimination against people living with HIV [58]. With the adverse, negative effects upon women that have been documented upon receiving an HIV test result, the enormous numbers presently being tested through the opt-out approaches in PMTCT programs raise serious ethical concerns. We argue that implementation of this testing approach should include partners to avoid venturing into a dangerous field which could lead women to choose not to disclose their status. A review carried out on rates barriers and outcomes of disclosure indicated that higher rates of women choose not to disclose (up to 86%). However it must be noted that majority of those who actually disclosed their HIV status reported supportive reactions from their partners [59]

In a context where there is the challenge of under-utilization of skilled attendance at birth services [60], confidence and trust are values at stake in the maternal health services. Testing pregnant women for HIV without their consent and without counselling to prepare them with a minimum of knowledge on how to live with either a HIV negative or a HIV positive status, can further diminish their confidence in the health providers and the health system, and can drive them away from vital maternity-related services. The weakened image of the health providers is part of a tendency also influenced by new public health management approaches [23].The challenges faced by health providers in implementing the new testing model may certainly to some extent, be attributed to the weak health systems in the region [61]. Sub Saharan Africa suffers a high burden of disease and the ratios of health workers per population are extremely low [62]. In contexts where the health system is grappling with limited resources, poor provider behaviour to clients has also been outlined among the many challenges facing HIV service delivery [61].

Unlike a VCT setting where very few seek testing services, the ANC environments are characterised by large numbers of the women as the main purpose is to test for mother and child related issues and not to test for HIV. Within such a scenario, health care providers may be overwhelmed by the high numbers of clients they receive for testing, hence influencing the manner in which the testing services are offered. This is an important part of the context in which health care providers work and coming mothers receive their HIV testing results, including little time for serious consideration of opt out processes of extended educational sessions. We thus argue that the 'blame' cannot primarily be placed on the health care workers. Our data e.g. reveals that pre and post test counselling are sufficiently observed at VCT. Strengthening the health system therefore seems to be vital if PITC implementation is to take place in the manner it was intended to. In this regard, involving lay counsellors in the testing and counselling services in these busy clinics as done in Botswana could be considered to reduce the burden on the health workers [39].

We argue that the challenges faced when implementing the provider initiated opt-out approach to HIV testing in many low income contexts, call for a renewed resource and rights-based momentum. Accountability for reasonableness, an ethically-based framework for fair decision-making and the fair priority-setting employed in the larger project of which this study is a part, draws our attention to the importance of trust in health systems [63]. The framework holds that securing trust in health systems will require approaches and arrangements that in a fundamental sense promote fairness and equity in a manner that respects human rights. From such a perspective implementing PITC for HIV in ways that compromises the right to obtain preventive information, and, for pregnant women, the right to opt-out or bypass consultation with their spouses are highly problematic. Trust between client and provider is critically important in an HIV context where vital but sensitive messages are to be communicated [18]. However, this cannot be secured through approaches implemented in a manner that threaten what has been considered as key in HIV testing; namely the possibility of receiving preventive information. The concern related to the extra burdens placed on women in vulnerable states of pregnancy adds to this scenario. We thus argue that HIV testing service delivery needs to be strengthened so that the trust and momentum that has been gained through the client-initiated services is retained. Respect for individual autonomy in HIV testing is essential as seen from the fundamental responsiveness goal of health systems [46,47].

Adopting a concurrent triangulation mixed methods design in this study reduces the potential weaknesses of using a single method. This design was chosen in order to capture both population testing exposure levels as well as people's experiences with the testing strategies. The data that was generated offered an opportunity to make inter-country comparisons, and in particular to compare setting differences with regard to levels of HIV testing exposure. The data collection tools in the three districts were similar in terms of indicator questions (the survey) and guidelines for the qualitative data collection. The additional qualitative component that was added in one of the districts (Malindi) elicited further information regarding experiences and perceptions of the provider initiated opt-out testing model. This added component strengthened the data as the additional FGDs and IDIs also enabled us to capture the perceptions and personal experiences of our informants at the community level, in addition to the facility-based information collected from all three districts.

As this additional study was carried out in 2009, a time when the PITC model had been implemented in most settings, it enabled us to capture experiences and perceptions of this new HIV testing model. However, conducting this study only in one district (Malindi) puts restrictions on the possibility for inter-country comparisons with regard to experiences and perceptions related to the provider initiated opt-out HIV testing models. Recruiting the respondents in this additional study (Malindi) from the community did not always reap direct experiences with the ANC testing; hence, further studies where respondents are recruited at the ANC settings could be considered. Our findings also suggest that the PITC model could have been practiced in a manner that seemed acceptable in the other two districts. This finding concurs with a recent study from Mbale district in Uganda [64]. Nevertheless, it is likely that, at the time, the data was collected in these settings (Kapiri Mposhi and Mbarali) the PITC opt-out model had not been scaled up extensively. We have reason to believe that the findings of this study are applicable in many other settings as similar concerns are raised in recent studies conducted in Kenya and Uganda, and concerns documented from Tanzania, on practices of testing pregnant women without their consent or proper counselling [50,52,53,65].

## Conclusion

The manner in which the new provider initiated opt-out HIV testing model is being implemented was found to have resulted in high neglect of pre- and post test counselling. This could hamper the inherent preventive potential in HIV testing contexts. Moreover, the lack of counselling was found to hinder people's ability to decline testing, hence raising serious ethical concerns. Furthermore, the new testing model appeared to add an unreasonable burden to pregnant women, in that the approach is implemented on a large scale primarily among women in the PMTCT programs, who are made responsible to recruit their spouses to go for a test. Further research is needed to: explore challenges from the perspective of health care workers and how to strengthen the health systems and in particular the ante natal clinics, so as to effectively implement the new testing model, assess how the opt-out testing approaches are implemented in other high HIV prevalence settings and to explore further how 'Accountability for reasonableness' or other rights-based frameworks can be drawn upon in ways that ensure that new strategies attempting to improve access HIV services are implemented in a manner that retains trust and a minimum of the patients' rights. Our findings indicates that there is an urgent need to reconsider the manner in which the opt-out approach to HIV testing is presently being implemented in order to protect each client's autonomy, to protect the right to access HIV prevention, and to protect pregnant women from an unreasonable additional burden.

## Competing interests

The authors declare that they have no competing interests.

## Authors' contributions

MKN was involved in conceptualising the objective of this paper, developing the interview guides for the second phase of qualitative interviews carried out in Malindi, conducting the interviews, analysing the quantitative and qualitative data, interpreting the findings and writing the manuscript. AB was involved in: developing REACT study tools, developing the interview guides for the additional study in Malindi, analysing and interpreting the qualitative findings and in extensive revision of the manuscript. EHS was involved in developing the REACT qualitative study tools, collecting the qualitative data in Mbarali, analysing the qualitative data and revising the manuscript. IKN coordinated the REACT qualitative studies in Kenya, reviewed the study guides for the second phase of qualitative interviews, was involved in analysing and interpreting the qualitative data and revised the manuscript. KF was involved in conceptualizing the REACT project as well as the objective of the present manuscript, he took part in the development of the design and data collection tools for the population-based survey, analysing the quantitative part of the study and revising the manuscript extensively. All authors read and approved the final manuscript.

## Note

^1 ^*s/he: in Swahili there is no separate word for she and he*

## Pre-publication history

The pre-publication history for this paper can be accessed here:

http://www.biomedcentral.com/1472-6963/11/87/prepub
